# Applications of pelvic neurophysiology testing in clinical practice

**DOI:** 10.1016/j.cnp.2026.01.008

**Published:** 2026-02-09

**Authors:** Jalesh N. Panicker, Sara Simeoni, Sarah Wright, Claire Hentzen, Prasad Malladi

**Affiliations:** a Department of Uro-Neurology, The National Hospital for Neurology and Neurosurgery, Queen Square, London, UK; b UCL Queen Square Institute of Neurology, Faculty of Brain Sciences, University College London, London, UK; c Department of Neurology, Dunedin Hospital, Te Whatu Ora - Health New Zealand, New Zealand; d Sorbonne University, GRC 01, GREEN Group of Clinical REsEarch in Neurourology, AP-HP, Hôpital Pitié Salpétrière, F-75013 Paris, France

**Keywords:** Anal sphincter EMG, Urethral sphincter EMG, Pudendal sensory evoked potentials, Bulbocavernosus reflex, Cauda equina syndrome, Multiple System Atrophy

## Abstract

Pelvic neurophysiology testing uniquely provides an evaluation of the sacral S2, 3 and 4 segmental somatic innervation. Since the tests are involved and some are invasive, a clear clinical question is essential in order to tailor the tests appropriately. Results should always be interpreted in conjunction with the clinical presentation and other investigation findings. The tests provide objective quantitative evidence for nerve injury and there are specific indications where these tests become of paramount importance such as the evaluation of suspected cauda equina syndrome, suspected MSA, chronic urinary retention, pelvic pain, sexual dysfunction and situations where MRI findings are inconclusive. Neurophysiology testing is a useful adjunct to clinical examination, and adds value to the diagnostic workup of patients presenting with neuro-urological disorders.

## Highlights

• Pelvic neurophysiology tests assess sacral somatic sensory and motor innervation.
• Tests are selected based on the referring clinical question.
• Evaluate suspected cauda equina injury, unexplained pelvic symptoms, unclear MRI.

## 1 Introduction

Pelvic neurophysiology has emerged within clinical neurophysiology as a validated and specialised set of investigations for the assessment of the sacral innervation. From modest beginnings in the 1950 s evaluating sphincter EMG and the bulbocavernosus reflex, advances in the field have now led to an array of tests capable of evaluating the integrity of the afferent and efferent somatic nerves derived from the sacral S2, S3 and S4 spinal cord segments innervating the pelvic floor muscles and sacral dermatomes, and to a lesser extent the pelvic splanchnic innervation. These techniques now find application across a range of specialities including urology, neurology, neurosurgery, physiatry, gastroenterology, urogynaecology and andrology. Information acquired from pelvic neurophysiology testing contributes to the diagnostic assessment of patients presenting with pelvic somatic and visceral symptoms with as diverse conditions as sacral cysts, parkinsonism and cauda equina syndrome. This review presents an overview of the different pelvic neurophysiology tests, and illustrates their practical utility in a clinical setting.

### 1.1 Pelvic innervation

**Fig. 1:**
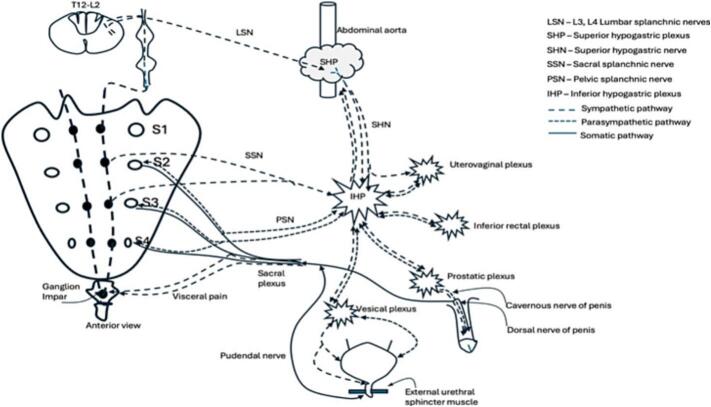
Sympathetic (LSN, SSN), parasympathetic (PSN) and somatic innervation of the pelvis. Postganglionic sympathetic nuclei are also present in the Superior hypogastric plexus and in the Inferior hypogastric plexus. Postganglionic parasympathetic nuclei are present near the end organ. The pudendal nerve carries the sensation from the external genitalia and provides motor innervation to the external urethral sphincter.

**Fig. 2:**
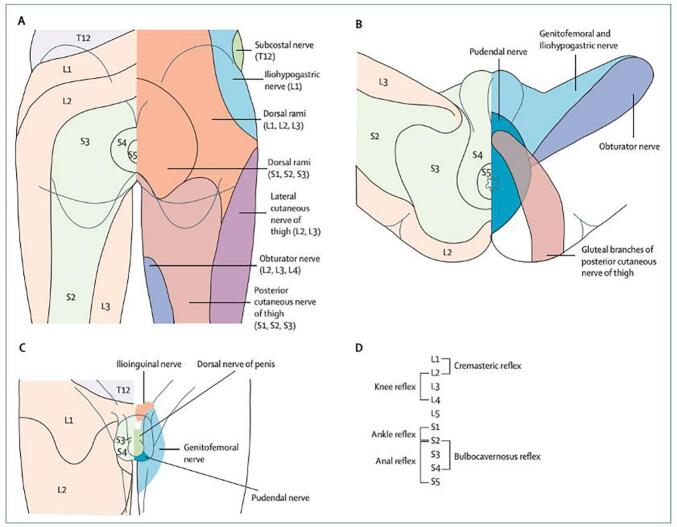
Illustration of the lumbosacral dermatomes and cutaneous nerve territories in the gluteal area and posterior thigh (A), perineum (B) and male external genitalia (C), and root values of lumbosacral cord mediated reflexes (D)(Panicker, Fowler and Kessler, 2015) (by permission from Elsevier).

The innervation of the pelvic floor and visceral organs is derived from sympathetic, parasympathetic and somatic nervous system, which is depicted in Fig. 1. The motor somatic innervation is derived from the anterior horn cells, particularly Onuf’s nucleus, at the S2, S3, and S4 sacral cord segments, and fibres descend through the sacral roots and the pudendal nerve to the pelvic floor muscles. Somatic sensory fibres innervate the sacral dermatomes, which are illustrated in Fig. 2 (Panicker, Fowler and Kessler, 2015), and the distal urethra and anal canal. Efferent parasympathetic fibres originate from the intermediolateral horn of the sacral spinal cord S2-S4 segment, and the preganglionic parasympathetic fibres travel through the sacral ventral roots and the inferior hypogastric plexus synapsing at ganglia situated near the pelvic viscera. Postganglionic fibres innervate the lower urinary tract, distal colon and external genitalia.

Preganglionic sympathetic fibres for the pelvic viscera originate from the intermediolateral horn of the spinal cord at the T12-L2 segments and descend through the L3 and L4 lumbar splanchnic nerves and synapse in the superior hypogastric plexus. Some of the preganglionic sympathetic fibres descend through the ventral root, white rami communicantes, sympathetic chain, and exit the sympathetic chain at the S2 and S3 foramen level, synapsing at the inferior hypogastric plexus.

The superior hypogastric plexus has both efferent and afferent sympathetic (nociceptive) fibres (Correia, 2010), and some parasympathetic fibres that may have ascended from the inferior hypogastric plexus (Darby, 2014). Hence, damage to the superior hypogastric plexus can result in ejaculatory and erectile dysfunction. This is particularly evident in patients with lower motor neuron lesions below L2, where there may be some preserved erections. However, due to a lack of sacral input, the erections would not be sufficient for penetration. A spinal cord lesion above T1 results in erectile dysfunction due to the lack of input to the superior hypogastric plexus.

Sympathetic fibres from the superior hypogastric plexus descend through the superior hypogastric nerve and merge with a complex mesh of sympathetic and parasympathetic fibres known as the inferior hypogastric plexus. The inferior hypogastric plexus has postganglionic sympathetic fibres, visceral afferent fibres, and pre- and post- ganglionic parasympathetic fibres. Since the micturition reflex, defecation reflex, and sexual afferent and efferent pathways transit through the inferior hypogastric plexus, injury results in incontinence, urinary retention and sexual dysfunction. Sympathetic and parasympathetic fibres branch from the inferior hypogastric plexus to form smaller plexuses near the end organs, such as the Inferior rectal plexus and uterovaginal plexus. The inferior hypogastric plexus provides sympathetic and parasympathetic input to the penis through the prostatic plexus and cavernous nerve. Erectile dysfunction after radical prostatectomy is therefore a well-known risk due to injury to the prostatic plexus.

## 2 Pelvic neurophysiology tests

### 2.1 Overview

Different tests have been established over the years to assess the pelvic somatic sensory and motor innervation. The choice of tests to perform depends upon the clinical question and the suspected level of neurological injury. These tests are summarised in Table 1.

**Table 1:** Queen Square pelvic neurophysiology protocol outlining tests used and the innervation they assess. BCR: Bulbocavernosus Reflex Study; dSEP: dermatomal sensory evoked potential; DCML: Dorsal column-medial lemniscus pathway; DNP.NCS: Dorsal nerve of penis sensory conduction study; SEP: sensory evoked potential; SSR: Sympathetic Skin Response.

	Pudendal sensory branches	Sacral sensory roots	Conus (S2-S4 sacral segments	DCML	Pudendal nerve motor branches	Sacral motor roots	Perineal cutaneous sympathetic innervation
Anal sphincter EMG			X		X	X	
Urethral sphincter EMG			X		X	X	
Pudendal nerve motor terminal latency					X		
BCR	X	X	X		X	X	
Pudendal SEP	X	X	X	X			
S2, S3 & S4 dSEP	X (specifically S4 dSEP)	X	X	X			
SSR							X
DNP.NCS	X						

### 2.2 Pudendal and sacral dermatomal sensory evoked potential studies

Mixed nerves such as tibial and median nerves consist of Aα motor fibres, fast conducting myelinated large-diameter Aα proprioceptive fibers (Type Ia) from muscle spindles, large diameter Aα proprioceptive fibers (Type Ib) from Golgi tendon organs, medium-diameter myelinated Aβ proprioceptive fibers (Type II) from secondary endings of muscle spindles, and thinly myelinated A δ and unmyelinated C- fibers. At the sub-motor threshold electrical stimulations, the tibial SEP shows low amplitude and prolonged cortical latency, presumably due to cutaneous stimulations (Cascino, 1988) via Aβ fibers, primarily associated with mechanoreception. At intermediate or strong intrinsic foot twitch stimulations, the tibial SEP latency reduces and amplitude increases, suggesting additional involvement of large-diameter myelinated proprioceptive fibers (A α − Type Ia and Ib). The tibial and median SEPs are also known as mixed SEPs (Cakmur, 1998, Essa et al., 2018, Tsai, 2012). As the name suggests, they enter the spinal cord from more than one root level and ascend through the dorsal column-medial lemniscus (DCML) pathway, reaching the somatosensory cortex, thereby providing objective evidence for abnormalities in the spinal cord, brainstem, or the cortex. Even though the mixed SEP technique is useful in the majority of cases, it is less sensitive in the evaluation of individual sensory root functions (Dikmen and Oge, 2013, Essa et al., 2018, Hakatifi, 1987), as single root abnormalities can be easily masked by fast conducting Aα proprioceptive fibers in other roots. In addition, apart from the ankle or wrist, the mixed SEP technique can not be easily applied in other areas of the body.

Segmental evoked potentials are cortical evoked potentials acquired by stimulating peripheral sensory nerves, such as the sural (Chiappa and Ropper, 1982, Perlik, 1986, Smith, 1988), superficial radial nerve (Grisolia and Wiederholt, 1980, Smith, 1988, Yiannikas et al., 1986) saphenous (Jain, 2015), dorsal penile / clitoris nerve (Williams, 2024) or perineal nerves (Jiamjunyasiri, 2023).

Since the peripheral sensory nerve conduction study is usually sufficient to assess the distal part of sensory nerves, the segmental evoked potential studies are particularly helpful in assessing the proximal part of the sensory nerves or sensory roots. Segmental evoked potentials are the only tool available in assessing proximal pudendal nerve lesions (Eisen et al., 1983, Kwon and Park, 2023, Ormeci, 2017, Yiannikas et al., 1983).

Segmental EPs, provides objective evidence for continuity of sensory nerve functions, however lack the ability to localise a lesion. Since pure sensory nerves lack Aα proprioceptive fibers (Type 1a & Type 1b), the amplitudes of segmental EPs are smaller than mixed SEPs (Behse, 1975, Burke, 1982). Segmental SEPs also suffer from the same disadvantage of mixed SEPs when it comes to the evaluation of individual sensory root functions.

Dermatomal sensory evoked potentials (dSEPs) are obtained by stimulating the cutaneous nerve fibres within the autonomous nerve zone of each dermatome exclusively innervated by the S2, S3 and S4 nerve roots, as shown in Fig. 2. The dSEP potentials follow the same somatosensory pathway of mixed SEPs or segmental SEPs but provide objective evidence for functional continuity of individual sensory roots. The normative values for sacral S2, S3 and S4 dermatome SEP studies have been established (Malladi, 2025). Even though dermatomal SEPs provide specific information about individual sensory roots, their amplitudes are smaller and latencies are prolonged compared to mixed SEPs (Cascino, 1988). Unlike mixed or segmental EP studies where the nerve trunk is stimulated, dSEPs are obtained by stimulating myelinated Aβ fibers in a small cutaneous area of a single dermatome. Hence, dSEP amplitude variability is significantly higher than mixed SEPs (Slimp et al., 1992). Due to their low amplitudes, dSEPs would not be an ideal choice for intraoperative monitoring.

In summary, mixed SEPs are useful in the evaluation of central demyelination and lesions that affect the spinal cord, brainstem or cortex. They also play an important role during spinal surgeries by continuously monitoring the sensory pathway integrity and preventing neurological damage. Segmental SEPs are helpful in evaluating lesions that affect the proximal part of sensory nerves, which are inaccessible for nerve conduction studies, such as a pudendal nerve lesion at the Alcock canal, Merlgia paraesthetica (Eltantawi, 2012), and radial nerve SEP in supraclavicular lesions (Synek, 1987). Dermatomal SEPs are helpful in the evaluation of individual sensory roots such as sacral S2, S3 and S4 dSEPs in Tarlov cysts (Hentzen, 2023), midline sacral meningeal cysts (Cabrilo, 2025), lumbosacral spinal stenosis and congenital scoliosis (Kraft, 2003, Snowden, 1992, Zhang, 2022). Hence, an appropriate SEP technique is needed to evaluate somatosensory pathway lesions.

### 2.3 Needle electromyography

Needle electromyography (EMG) is the most widely used test in clinical neurophysiology for evaluating myopathic conditions and both active and chronic denervation changes in muscles. EMG of the pelvic floor muscles is useful for assessing lesions that affect the anterior conus, ventral sacral roots, sacral plexus, and pudendal nerve. However, since these muscles receive innervation from the S2-S4 sacral roots, the test is unable to assess the nerve roots individually.

Anal sphincter EMG is performed by inserting the needle electrode at the left and right lateral aspects of the anal canal (9 and 3o'clock positions) typically near the mucocutaneous junction, with the patient in the left lateral decubitus position. Unlike the muscles of the upper and lower limbs, the external anal sphincter muscle is tonically active. Therefore, evaluating individual motor unit action potentials (MUAPs) in this muscle is challenging. Furthermore, spontaneous activity can often be masked by the tonic activity of the anal sphincter muscle and may only be recorded when anal tone is reduced, as seen in Multiple System Atrophy (MSA). Direct injury to the anal sphincter can occur following obstetric trauma or anorectal surgery, including haemorrhoidectomy. Consequently, the presence of chronic denervation in such individuals does not necessarily indicate sacral root or pudendal nerve pathology. The external urethral sphincter is similarly tonically active, and EMG is most commonly performed in the context of a diagnostic assessment for women presenting with chronic idiopathic urinary retention. The urethral sphincter EMG test is typically performed using a local anaesthetic applied at the needle insertion site, which is approximately 5 mm anterior or lateral to the external urethral meatus. The bulbocavernosus muscle shows minimal activity at rest, making it easy to record spontaneous activity when there is a suspicion of an acute lesion involving the conus or sacral motor roots, or the pudendal nerve.

### 2.4 Bulbocavernosus reflex study

The bulbocavernosus reflex (BCR) is a standard clinical bedside test used to evaluate the integrity of the S2-S4 sacral segment of the spinal cord, including afferent input from the penis or clitoris and efferent output to the bulbocavernosus muscle or anal sphincter. The presence of a visible contraction of the anal sphincter (anal wink) following a firm squeeze of the glans of the penis or clitoris suggests normal conus function. However, eliciting a robust anal wink requires the patient’s cooperation and effective execution of the penile or clitoral squeeze, which can sometimes be challenging. The neurophysiological recording of the BCR provides an objective assessment of the functions of the conus and cauda equina (Podnar, 2009). BCR responses recorded from the bulbocavernosus muscle using a concentric needle are both reliable and reproducible. The exact mechanisms underlying the R1 and R2 responses remain unclear. However, the R1 response is widely believed to be oligosynaptic, given its relatively short latency (Vodusek and Janko, 1990). The R2 response is considered a polysynaptic response involving afferent Aδ fibres (Amarenco, 2002, Vodusek et al., 1983). The afferent pathway of the BCR comprises not only Aα sensory fibres but also Aβ and Aδ fibres conveying pressure and vibration sensations from the glans and shaft, which are partly responsible for bladder, bowel, and sexual functions.

Typically, the BCR response comprises an R1 response with a latency of approximately 30 ms and an R2 response with approximately double that of R1. Although R1 responses often accompany R2 responses, this is not always observed in healthy subjects. The amplitude of BCR responses is significantly influenced by the placement of needle or surface electrodes. Because R2 responses are prone to habituation, for all clinical purposes R1 latency is the only parameter used to interpret BCR results. The upper limit of R1 latency is 40 ms in males (mean + 2SD) and 45 ms in females (Granata, 2012, Podnar, 2011, Tankisi, 2016, Vodusek, 1983).

Although the absence of an R1 response is highly suggestive of lesions of the conus or cauda equina, its presence does not rule out a partial lesion (Blaivas, Zayed and Labib, 1981). BCR responses have been shown to be normal in patients with functional erectile dysfunction (Bird and Hanno, 1998). Huang (2018) suggested that the BCR test can be used to differentiate Multiple System Atrophy (MSA) from Parkinson’s disease (PD), however in this study and subsequent ones (Niu, 2022, Pan, 2020), R1 latencies exceeded 60 ms, falling within the R2 latency range, raising questions about the accuracy of R1 latency measurement. Hu (2023) observed that the specificity of the BCR test alone for differentiating MSA from PD is no more than 40%. Given the very limited number of studies available, caution is needed when using the BCR test alone to differentiate MSA from PD.

### 2.5 Dorsal penile nerve conduction study

The dorsal nerve of the penis (DNP) is the terminal sensory branch of the pudendal nerve, innervating the distal urethra, glans, and shaft of the penis. These travel over the dorsal surface on either side of the shaft respecting the midline. They are responsible for sensations on their respective sides but never cross the midline. Further distally, the DNP divides into multiple smaller branches, supplying the dorsolateral skin before reaching the glans. The nerve remains superficial on the shaft and travels deep into the epithelium upon reaching the glans (Yang and Bradley, 1998). For this reason, sensory responses are much more easily recordable from the shaft than from the glans (Tunçkol, 2023). In addition, the distribution of sensory fibres in the DNP differs between the shaft and glans (Tunçkol, 2023). More than fifty per cent of the DNP consists of unmyelinated fibres. Unmyelinated and thinly myelinated fibres in the DNP predominantly supply the glans, whereas myelinated fibres innervate the shaft. The Aδ fibres in the DNP are stimulated during an erection as the surface area of the glans increases, and the epithelium undergoes stretching. Consequently, sexual responses increase with the recruitment of a larger number of cutaneous receptors and small nerve fibres.

**Fig. 3:**
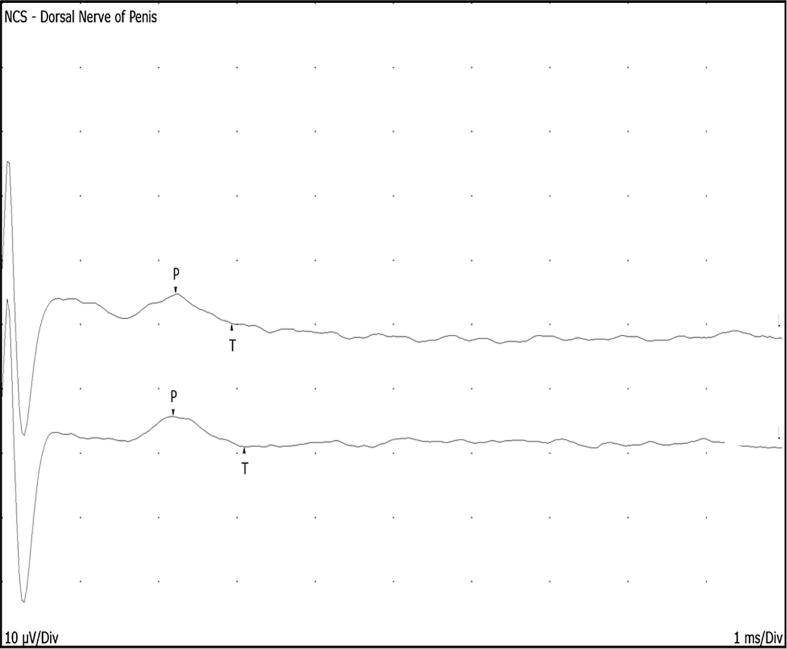
Dorsal nerve of the penis sensory conduction study demonstrating a normal response.

The nerve conduction study technique assesses large-diameter myelinated fibres. The conventional nerve conduction study of the DNP involves elongating the penis using a traction device and stimulating the nerve orthodromically (Clawson and Cardenas, 1991, Kaneko, 2002, Kiwamoto, 1988). However, the technique employed at Queen Square resembles nerve conduction studies conducted in the limbs, where the active electrode is positioned on the distal shaft, avoiding contact with the glans, while the reference electrode is placed on the ventral distal shaft. The DNP is stimulated at the root of the shaft with a paediatric stimulator for all patients. This technique is well-tolerated, and the sensory morphology closely resembles the median nerve sensory response, although it exhibits a much shorter peak latency and amplitude, as illustrated in Fig. 3.

Our experience from over 60 patients suggests that the mean peak latency is 1.9 ms ± 0.4 SD, regardless of penile length. We consider the absence of a response to be the abnormal criterion, rather than reduced amplitude or prolonged peak latency.

### 2.6 Other tests

#### 2.6.1 Sympathetic skin response

**Fig. 4:**
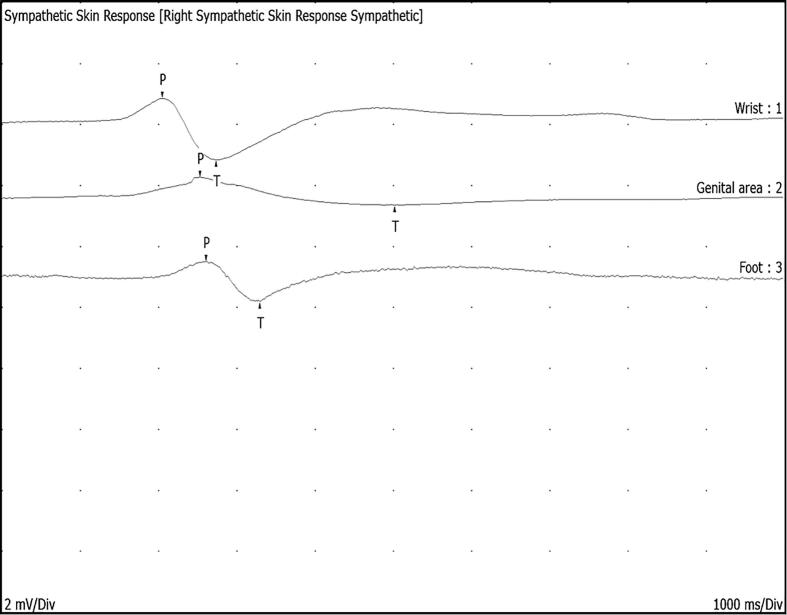
Sympathetic skin response recorded from the wrist, genital area and from the foot after stimulating at the ipsilateral wrist.

The sympathetic skin response (SSR) test has been utilised in clinical practice for over five decades to evaluate sympathetic autonomic fibres. An electrical stimulation at the wrist or ankle can reliably elicit the SSR response from the upper and lower limbs and the perineum, and its typical waveform is illustrated in Fig. 4. Although stimulation in the SSR test travels through the afferent somatic sensory pathway, the efferent pathway is through the postganglionic unmyelinated C-fibres to eccrine sweat glands (Arunodaya and Taly, 1995).

Abnormalities in SSR are well-documented in peripheral neuropathies (Liu, 2024, Valls-Sole et al., 1991) and mononeuropathies (Mondelli and Aretini, 2024). The SSR has been demonstrated to be abnormal in MSA (Knezevic and Bajada, 1985, Ravits, 1986) with the latencies deteriorating as the disease advances (Shindo, 2020). Furthermore, SSR studies have shown potential in differentiating patients with MSA-P from PD in the early stages (Xie et al., 2024). The presence or absence of SSR following spinal cord injury (SCI) depends on the level of the lesion and the site of stimulation. Palmar responses in the SSR test can be recorded following lesions below T3, while palmar and plantar SSRs can be documented after lesions below T12. Plantar SSRs may be recorded following pudendal nerve stimulation in patients with complete cervical and thoracic SCI; however, they are absent in individuals with lesions at L1 or further caudally (Reitz, 2002). As mentioned previously, the sympathetic outflow to the genital area follows a more complex route than that to the bladder, and studies evaluating SSR in patients with genitourinary and sexual dysfunctions have yielded mixed results (Chen et al., 2024, Coxon, 2023, Seçıl, 2007).

#### 2.6.2 Pudendal nerve terminal motor latency (PNTML) testing

The inferior rectal nerve is a mixed nerve that is the most proximal branch of the pudendal nerve exiting Alcock’s canal. The branch innervates the external anal sphincter and is responsible for sensations at the anal opening and surrounding skin. The nerve is accessible only at the level of the ischial spine and hence can be studied only in a short segment of less than 5 cm. However, distal motor latencies are unreliable for shorter distances less than 7 cm (Preston and Shapiro, 2012) due to baseline distortion and depolarisation under reference electrodes. Stimulating and recording points cannot be visually verified when using St Mark’s electrodes in PNTML studies, and more current is often required to stimulate the nerve. Technical expertise is also required in order to obtain a good PNTML response while maintaining the patient’s tolerance to the test. In addition, compounding factors such as age, childbirth, menopause, and surgical procedures in relation to the anal canal can influence the anal sphincter tone and function. These technical and physiological factors make PNTML more challenging to interpret and opens a discussion on the reliability and clinical usefulness of this test. Initial publications suggested a promising role for this test in the evaluation of faecal incontinence and pudendal nerve dysfunction (Jones, 1987, Kiff and Swash, 1984). However, several large-scale studies have shown contradictory results (de Aguiar Cavalcanti, 2013, Kwakye and Maguire, 2021, Remes-Troche and Rao, 2008, Saraidaridis, 2018) which have generally discouraged the use of this test.

#### 2.6.3 Quantitative sensory testing

Small-fibre testing has become well established over the past five decades (Backonja, 2009, Gruener and Dyck, 1994, Rolke, 2006). QST comprises seven tests, including Thermal Threshold Testing (TTT), mechanical detection threshold, and mechanical pain threshold, and enables the recording of thirteen parameters, such as cold detection threshold, warm detection threshold and mechanical pain threshold, thereby providing a comprehensive assessment of small-fibre function (Rolke, 2006). However, performing all QST modalities in routine clinical practice is not feasible. Hence, many centres restrict QST to a single modality, most often TTT, to broadly assess small-fibre function in the lower and upper limbs (Raasing, 2023, Sheen, 2018). When objective evidence of small-fibre loss is required, a skin biopsy is used to assess the loss of small-diameter nerve fibres at the cutaneous level (Ebenezer, 2007, McArthur, 1998). A skin biopsy is usually taken from the distal leg, and in some cases, a second sample is taken from the lateral thigh to assess length-dependent neuropathy.

Large-fibre testing, including pudendal SEP, needle EMG, and pudendal nerve conduction studies, is well established in the pelvic region. However, small-fibre testing in the pelvic region is yet to be established. Performing all small-fibre tests on the external genitalia is quite challenging, and obtaining a skin biopsy from this area is not a practical option. Even a single small-fibre test, such as TTT, poses significant practical challenges in the external genitalia. The standard TTT thermode probe cannot be used on small areas such as the glans of the penis or the clitoris, and therefore normative values generated from the foot or hand cannot be applied to the external genitalia.

Specialised small-area thermode probes were used in a few studies to assess small-fibre functions in the mouth (Naganawa, 2015) and over the external genitalia (Burke and Lowenstein, 2016, Gruenwald, 2007, Vardi, 2000), demonstrating the feasibility of extending small-fibre testing to specialised areas. Large studies researching small-fibre testing in the pelvic area are still lacking due to the unavailability of miniature QST equipment and a lack of awareness and coordination among healthcare providers who manage pelvic complaints. Until QST is standardised in the pelvic area, chronic pain, allodynia, hyperalgesia, hyperaesthesia and hypoaesthesia can be assessed only clinically (Chen et al., 2019, Hovaguimian and Gibbons, 2011, Ilaka, 2024, Mozafarpour, 2022).

## 3 Clinical applications of pelvic neurophysiology tests

### 3.1 Cauda equina syndrome

Compression to the cauda equina occurs most commonly following disc prolapse, trauma and neoplasms and results in devastating lower urinary tract, bowel and sexual dysfunction. Denervation changes can be observed in the pelvic floor muscles as early as 2 to 3 weeks following injury, and neurophysiology testing is useful to assess ongoing somatic nerve damage by the presence of spontaneous denervation activity in the EMG. Quantitative EMG can demonstrate evidence for chronic denervation characterized by reduced interference pattern and polyphasic MUAPs with prolonged duration and abnormally increased amplitudes. Studies have shown a correlation between neurophysiological changes and the severity of sexual dysfunction (Podnar, Oblak and Vodušek, 2002). Anal sphincter EMG and BCR have high sensitivity and negative predictive value in confirming and excluding sacral root lesions (Podnar, 2014). Absence of the BCR during the acute stage of injury has been shown to prognosticate poorer recovery of bladder and sexual functions. Conversely, the presence of a BCR response with either normal or prolonged latency has been associated with recovery in bowel, bladder and sexual functions (Lavoisier, 1989, Lee et al., 2017). Pelvic neurophysiology tests are able to accurately assess the extent of damage across the different sensory and motor sacral roots, particularly helpful when injury has been partial. Case vignette 1 illustrates a case of cauda equina syndrome.

**Case vignette 1.** A case of cauda equina injury.

A female in her 40 s presented with a 10-year history of low back pain with alternating lower limb sciatica. She developed a 1-month history of worsening left leg sciatica and a 3-week history of perineal and perianal numbness and impaired sensations of bladder fullness. MRI revealed a large extruded disc fragment and lumbar canal stenosis at L5/S1, and she underwent emergency decompression and discectomy for cauda equina compression. Following the surgery, her sciatica improved significantly. However, she was experiencing persistent perineal numbness and was found to be retaining urine in the bladder after voiding.

**Fig. 5:**
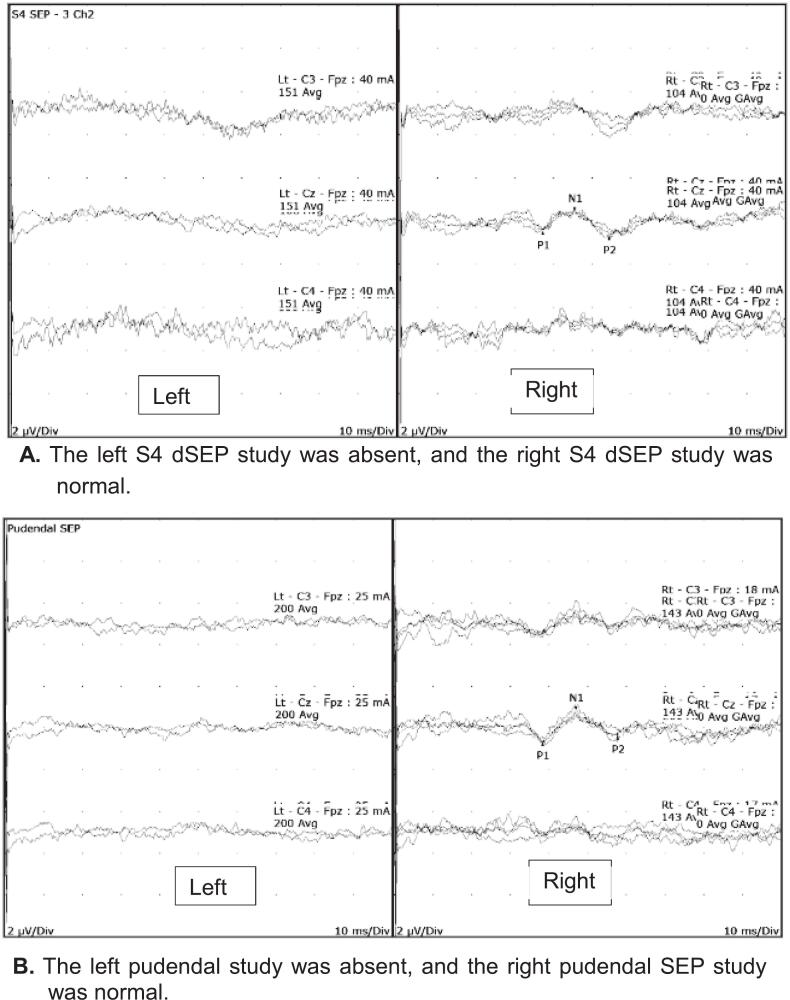
A. The left S4 dSEP study was absent, and the right S4 dSEP study was normal. Fig. 5B. The left pudendal study was absent, and the right pudendal SEP study was normal.

She was referred for pelvic neurophysiology testing with the question whether there was a neurological cause for her perineal symptoms and to assess the extent of sacral root injury. Her neurological examination revealed sensory impairment to pinprick over the S2-S3 dermatomes on the left side and the S4 dermatome bilaterally. Anal sphincter tone was reduced. Neurophysiology tests were abnormal as shown in Table 2 as illustrated by the left S4 dSEP and pudendal SEP tests (Fig. 5A and 5B).

**Table 2:** Summary of pelvic neurophysiology findings in case vignette 1.

Test	Right	Left
Tibial SEP	Normal	Abnormal (5.9 ms delay)
S2 dSEP	Normal	Absent
S3 dSEP	Normal	Absent
S4 dSEP	Normal	Absent
Pudendal SEP	Normal	Absent
BCR	Normal	Absent
Anal sphincter EMG	Normal	Abnormal −Active denervation changes

These findings were consistent with injury to the cauda equina on the left side.

Pelvic neurophysiology studies are particularly useful to evaluate the integrity of the cauda equina in patients who present with back pain and urogenital and bowel dysfunction consistent with cauda equina syndrome, where MRI does not demonstrate significant compression of the sacral roots. This can occur following neuropraxia or an ischemic event resulting in microstructural injury where patients may present with cauda equina syndrome without evidence for compression in MRI. An example is provided in Case vignette 2.

**Case vignette 2.** A case of cauda equina syndrome, without evidence for sacral root compression in MRI.

A male in his 60 s experienced acute onset back pain and a feeling of “giving way” after pulling out a peg from the ground, and within hours began to experience saddle numbness and went into urinary retention. He developed constipation, and subsequently problems with sexual performance. MRI lumbosacral spine revealed degenerative changes throughout the spine, however there was no evidence for compression of the sacral roots (cauda equina) or conus medullaris. A urological evaluation showed a normal-sized prostate gland. The urodynamic study showed detrusor underactivity in the pressure flow study.

**Fig. 6:**
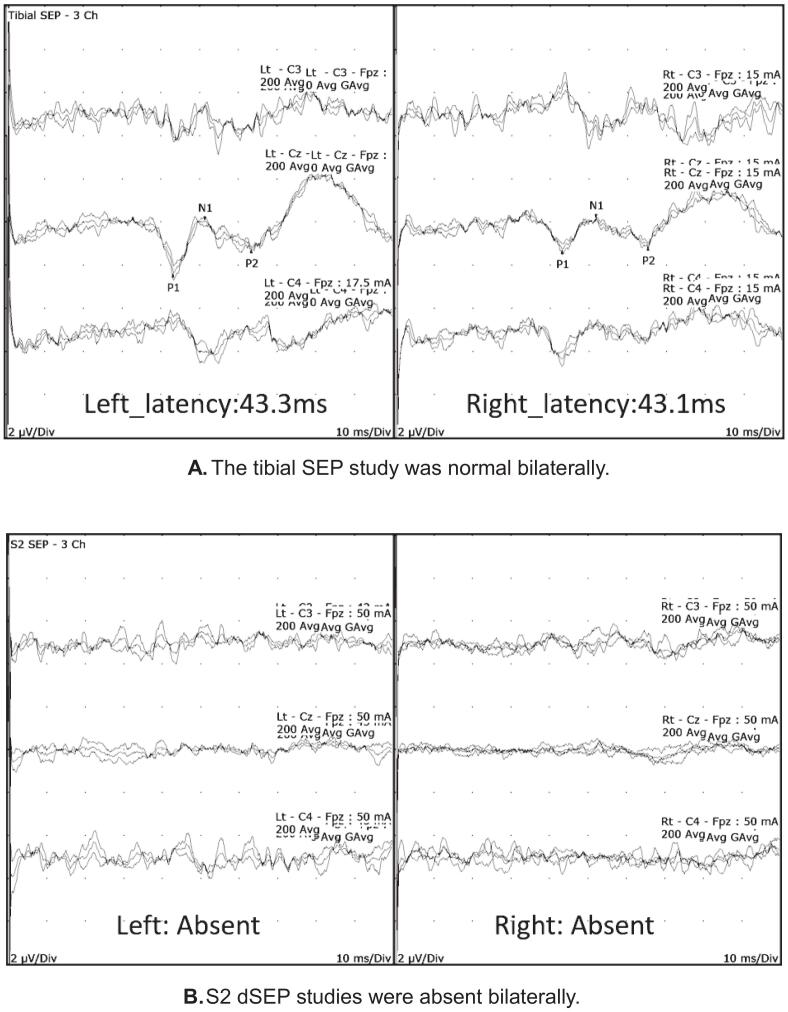
A. The tibial SEP study was normal bilaterally. Fig. 6B. S2 dSEP studies were absent bilaterally.

He was referred for pelvic neurophysiology testing with the question whether there was a neurological cause for his pelvic symptoms. The neurological examination revealed normal power and deep tendon reflexes in his lower limbs. Anal sphincter tone was impaired and the anal reflex and BCR were absent. Sensory impairment was noted over the S2, S3 and S4 dermatomes bilaterally. The neurophysiology tests were abnormal (Table 3) and findings in the tibial SEP and sacral dSEP tests are illustrated in Fig. 6A and 6B.

**Table 3:** Summary of pelvic neurophysiology findings in case Vignette 2.

Test	Right	Left
Tibial SEP	Normal	Normal
S2 dSEP	Absent	Absent
S3 dSEP	Absent	Absent
S4 dSEP	Absent	Absent
Pudendal SEP	Abnormal	Abnormal
BCR	Absent	Absent
Anal sphincter EMG	Abnormal with chronic denervation changes	Abnormal with chronic denervation changes

These findings were consistent with extensive injury of the cauda equina with bilateral involvement of the sacral roots, and correlated with the clinical examination.

### 3.2 Evaluating lumbosacral MRI findings of uncertain clinical relevance

Patients undergo lumbosacral MRI for different indications, and sometimes there are findings which are assumed to be incidental not relevant to symptoms, and may not even be mentioned in the MRI report. These include fatty filum terminale and sacral cysts (Klepinowski et al., 2021, Uchino et al., 1991). The incidence of fatty filum terminale or lipomas of the terminal filum has been reported to be around 4% in a study reviewing more than 50,000 patient records (Cools, 2014). However if these lesions compress the sacral roots, they can cause symptoms such as saddle numbness or pain, urogenital and bowel symptoms in which case they are of great relevance and pelvic neurophysiology can provide objective evidence of sacral root dysfunction. Certain MRI characteristics have been associated with symptomatic cases of tethered cord requiring surgery such as low-lying conus medullaris and fat close to the conus medullaris (Bulsara, et al., 2004, Cools, 2014). Furthermore, it would be important to objectively assess sacral root functions in such patients who have co-existing pelvic visceral organ co-morbidities such as pelvic organ prolapse before deciding upon neurosurgical or urological interventions.

Sacral cysts can present with back pain, perineal pain and sensory loss, and bladder, bowel and sexual dysfunction, and pelvic neurophysiology testing is able to accurately identify sacral nerve root damage (Cabrilo, 2025, Hentzen, 2023, Mieke, 2017). However despite promising outcomes reported in neurosurgical studies, pelvic symptoms do not always correlate with the location of cysts (Dowsett, 2018). In a series of 65 patients with pelvic symptoms and sacral Tarlov (perineurial) cysts, the most common sacral cyst, abnormal neurophysiological findings were observed in 57% of patients, primarily affecting the sensory innervation, despite the sensory clinical examination being normal in most of these patients. Furthermore, a negative association was found between overactive bladder symptoms and abnormal neurophysiology findings, suggesting that urinary storage symptoms were occurring independently (Hentzen, 2023). Given these uncertainties, a comprehensive pelvic neurophysiology testing including an objective evaluation of the sensory innervation with pudendal and dermatomal SEPs, motor innervation with anal sphincter EMG, and the bulbocavernosus reflex can provide valuable insight into the impairment of sacral nerve root functions. Midline sacral meningeal cysts are seen often than Tarlov cysts, however are more likely to cause sacral nerve root damage (Cabrilo, 2025). Case vignette 3 illustrates a case of gluteal pain and perineal numbness with an abnormal MRI lumbosacral spine.

**Case vignette 3.** A case of back pain with abnormal findings in the MRI lumbosacral spine of uncertain clinical relevance.

A female in her 40 s fell down a flight of steps a year ago and presented with low back pain, and pain in the perineum that extended down both lower limbs bilaterally more on the right side. She began to experience urinary hesitancy, and the urine flow became intermittent. She also began to experience constipation. The post void residual volume was normal. MRI lumbosacral spine was abnormal and demonstrated a large multiseptate Tarlov cyst arising in relation to the termination of the thecal sac and scalloping the posterior aspect of the sacrum and the sacral lamina. The cyst extended from S1 down to S4 segment.

**Fig. 7:**
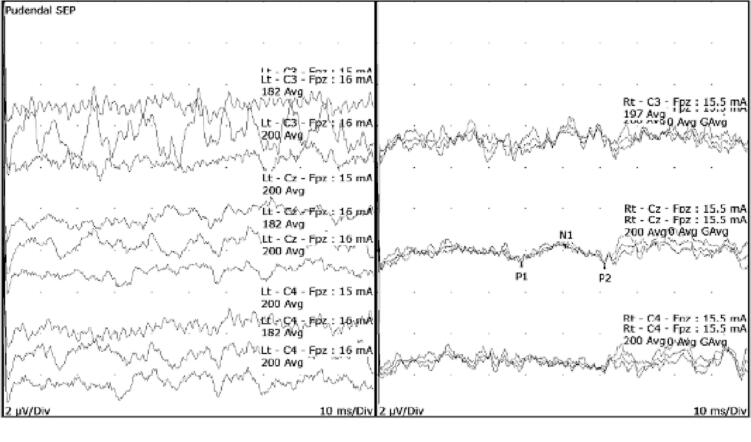
The pudendal SEP was normal on the right but absent on the left.

She was referred for pelvic neurophysiology testing with the question whether the changes seen in MRI were causing sacral root injury. Her neurological examination revealed reduced sensations to pinprick over the perineum on the right side involving the clitoris, labia majora and minora and anal opening, extending to the gluteal area. Anal sphincter tone was normal. The rest of her neurological examination was normal. The tests were abnormal as shown in Table 4, and the pudendal SEP findings are illustrated in Fig. 7.

**Table 4:** Summary of pelvic neurophysiology findings in case vignette 3.

Test	Right	Left
Tibial SEP	Normal	Normal
S2 dSEP	Normal	Normal
S3 dSEP	Absent	Normal
S4 dSEP	Absent	Absent
Pudendal SEP	Normal	Absent
BCR	Normal	Absent
Anal sphincter EMG	Normal	Normal

These tests were consistent with involvement of the S3 and S4 roots bilaterally, which can occur in patients with sacral Tarlov cyst.

### 3.3 Diagnostic assessment of atypical parkinsonian syndromes

MSA is often a differential diagnosis when patients present with atypical parkinsonism, and is a progressive neurodegenerative disease characterized by cardiovascular and urogenital autonomic failure, and parkinsonian, cerebellar and pyramidal dysfunction. Due to variability in the clinical presentation, it is often challenging to distinguish MSA from PD, particularly in the early stages of presentation (Fanciulli and Wenning, 2015). Urogenital symptoms are commonly reported in MSA, and lower urinary tract dysfunction can be the initial manifestation of MSA in 18% of patients (Sakakibara, 2019a). The lumbosacral spinal cord is commonly affected in this condition, and autopsy studies have demonstrated degeneration in Onuf’s nucleus, a population of neurons in the anterior horn of the S2-4 segments (Konno, 1986). Pathological changes have been shown to start early in the disease’s course (Stemberger, 2010) and some patients may present initially with chronic urinary retention and sexual dysfunction suggesting a sacral cord onset of disease (Panicker, 2020).

Neurogenic changes have been demonstrated in muscles innervated by Onuf’s nucleus, particularly the external anal sphincter (Beck, Betts and Fowler, 1994). The prevalence of neurogenic changes varies between 52% and 93% (Kirchhof, 2003, Pellegrinetti, 2003, Sakakibara, 2019, Todisco, 2022, Yamamoto, 2005) depending upon the patient cohort and neurophysiological parameter evaluated. Studies have shown that MUAP duration and the presence of spontaneous activity are the most reliable criteria in distinguishing MSA from other similar conditions, such as PD (Palace et al., 1997, Paviour, 2005, Qiu, 2019, Rodi et al., 1996, Tison, 2000, Yamamoto, 2005).

There are challenges in interpreting the findings of anal sphincter EMG when trying to differentiate MSA from PD. The test will be normal if the Onuf’s nucleus has not been significantly affected yet and in one case series, abnormalities were seen in only 52–62% of cases in the first year after disease onset, which increased to 83% by the fifth year (Yamamoto, 2005). Furthermore, an abnormal result is not pathognomonic for MSA and neurogenic changes have also be reported in other disorders such as longstanding PD (Libelius and Johansson, 2000, Paviour, 2005), Progressive Supranuclear Palsy (Valldeoriola, 1995, Winge, 2010), Dementia with Lewy Body (Tateno, 2015), Machado–Joseph disease, (Sakakibara, 2004) and Spinocerebellar ataxia type 6 (Tateno, 2012), suggesting that the Onuf’s nucleus can be affected in other disorders as well. However the degree of abnormalities are generally reported to be mild in these conditions, and occur later in the disease course. The utility of EMG in differentiating MSA from PD diminishes significantly over time, specially after five years of symptom onset (Libelius and Johansson, 2000, Paviour, 2005).

**Fig. 8:**
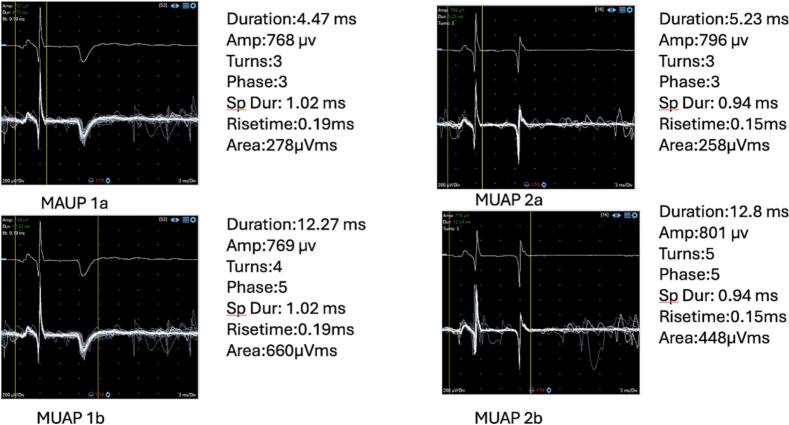
MUAP 1a and 2a are derived from a single study demonstrating normal mean durations when satellite potentials are excluded. However, the mean durations become abnormal when satellite potentials are included, as illustrated in MUAP 1b and 2b. The study was repeated after two years, and the EMG showed no deterioration; there were also no significant changes in the patient’s clinical symptoms.

The criteria used to define abnormality also affects the accuracy of the test in differentiating MSA from PD (Gilad, 2001, Linder, 2012, Schwarz, 1997). A core issue is regarding the duration of MUAPs and whether to include or exclude satellite potentials—low-amplitude, delayed discharges that are time-locked to the MUAP and occur after it, with variable latency. Most published studies on EMG in MSA are retrospective studies that utilise data collected over several years in patients where the diagnosis is secure. Hence, the nature of MUAP potentials in the early stages of MSA remains unclear. Conventional wisdom suggests that a MUAP cut-off duration of 10 ms should be applied. Even though alternative or supplementary criteria have been suggested, such as adding polyphasic potentials, increased amplitude of more than 1 mV, or a percentage increase in either duration or polyphasics between 20 and 50% to the mean duration criteria, the widely used definition is a mean duration of MUAP greater than 10 ms to differentiate MSA from PD (Beck et al., 1994, Libelius and Johansson, 2000, Niu, 2022, Valldeoriola, 1995). However, when this criterion is applied to a case of suspected MSA, the outcome varies from completely normal to completely abnormal depending upon where the end cursor is placed, which introduces selection bias, as shown in Fig. 8. In this specific case, the test was repeated after two years and there were no differences seen in the EMG findings. At Queen Square we use the automated MUAP analysis mode and measure the MUAP duration without satellite potentials.

Similar changes of denervation and reinnervation have also been recorded from the external urethral sphincter EMG (Beck et al., 1994, Eardley, 1989, Qiu, 2019). However external anal sphincter EMG is more often performed as this test is better tolerated by the patients, is technically easier to perform and has published criteria that define abnormalities. In contrast to the sphincters, the bulbocavernosus muscle is not tonically active and therefore the muscle can be sampled to assess spontaneous activity. The BCR also assesses Onuf’s nucleus, and a prolonged latency or absent response has been demonstrated in patients reporting urogenital symptoms (Cai, 2017, Huang, 2018, Niu, 2022). Abnormalities in BCR was found to differentiate MSA from PD with a sensitivity of 24% and specificity ranging from 91 to 94%(Cai, 2017, Huang, 2018).

**Case vignette 4.** A case of atypical parkinsonism with diagnostic uncertainty .

A female in her late 50 s presented with a rapidly progressive parkinsonian syndrome of 4 years duration and was found to have orthostatic hypotension 2 years ago. In the last couple of years, she was experiencing urinary urgency and urgency related incontinence, nocturnal polyuria, urinary hesitancy and an intermittent stream. Her postvoid residual volume was 55 mL, which was normal. She was diagnosed with pelvic organ prolapse a few years ago, though this was mild. She sustained a significant tear during one of her 3 normal deliveries several years ago. MRI scan showed slightly asymmetric mineralisation of the posterior putaminal region and mild putaminal volume loss on the right side. There was no evidence for disproportionate atrophy of the brainstem or cerebellum. DaTscan showed reduced tracer accumulation in both putamina, worse on the right.

**Fig. 9:**
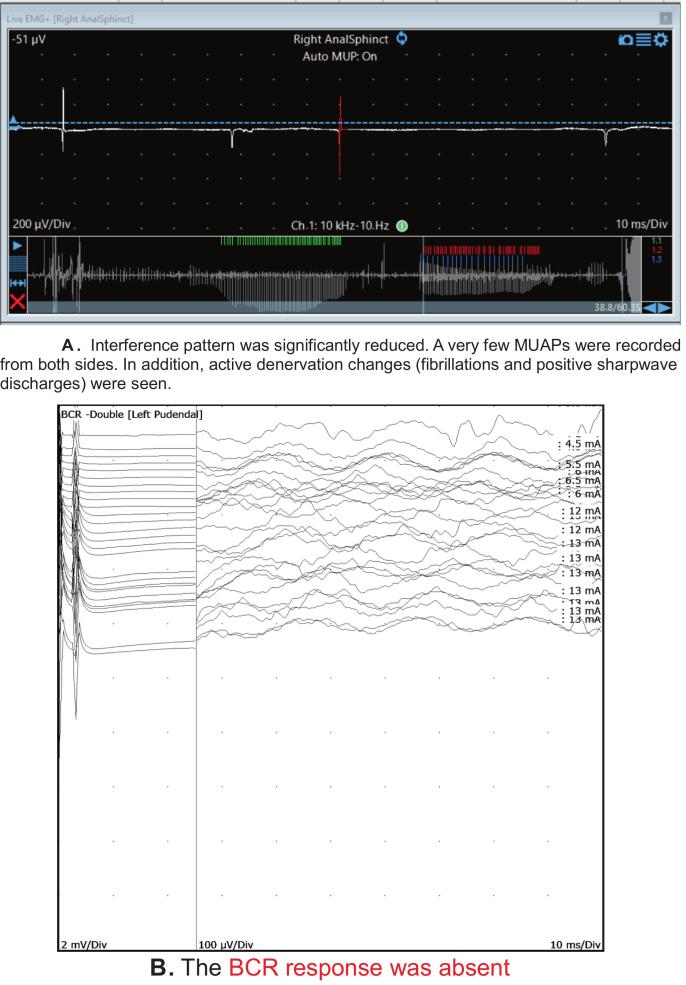
A. Interference pattern was significantly reduced. A very few MUAPs were recorded from both sides. In addition, active denervation changes (fibrillations and positive sharpwave discharges) were seen. Fig. 9B. The BCR response was absent.

She was referred for pelvic neurophysiology testing with the question whether there was any evidence for involvement of Onuf’s nucleus occurring in the context of an atypical parkinsonian syndrome. Her neurological examination revealed facial hypomimia and broken pursuit eye movements bilaterally. She had anterocollis, and bilateral rigidity and bradykinesia; there were no cerebellar signs. A stooped posture was noted and she required a walking frame because of a shuffling gait. Her pelvic neurological examination revealed reduced anal sphincter tone and squeeze, and the anal reflex was absent. Sacral dermatomal sensations were normal. The neurophysiology tests were abnormal and the findings of the BCR and external anal sphincter EMG are illustrated in Fig. 9A and 9B.

The findings were consistent with involvement of Onuf’s nucleus, and correlating with her clinical examination and investigation findings the likely clinical diagnosis was MSA-P.

### 3.4 Evaluation of young females presenting with chronic idiopathic urinary retention

Chronic idiopathic urinary retention/Fowler’s Syndrome is uncommon in females, and abnormal EMG activity was first demonstrated in the external urethral sphincter in the 1980 s by Clare Fowler and colleagues (Fowler, 1988). Patients with Fowler’s syndrome typically present in chronic urinary retention of more than a litre; however, they lack the sense of urinary urgency or bladder pain generally associated with such large volumes. When performing intermittent self-catheterisation, patients often report an abnormal sensation as if the catheter is being “gripped” when removed. A significant proportion of patients report polycystic ovaries (Osman and Chapple, 2014). Urethral sphincter EMG, urethral pressure profilometry and urethral sphincter volume have been demonstrated to be abnormal, suggesting that the condition is due to a primary disorder of urethral sphincter relaxation (Wiseman, 2002).

Spontaneously occurring fast-firing spontaneous discharges can be recorded from the external urethral sphincter EMG, and these include complex repetitive discharges (CRD), which are bizarre repetitively-firing highly symmetric bursts of variable frequency that have a characteristic sound similar to a helicopter over the loudspeaker of the EMG machine. Complex repetitive discharges may have a prominent decelerating component, called decelerating bursts, which have a myotonia-like quality (Fowler, 1988, Panicker et al., 2018). The frequency and rate of deceleration of these bursts have not been studied and they are recognisable by their characteristic sound over the loudspeaker, having a quality similar to the underwater recording of whales.

The mechanisms responsible for urinary retention in this condition remains unclear. Complex repetitive discharges are non-specific findings of nerve injury and could suggest an increase in muscle fibre grouping from reinnervation and ephaptic transmission between muscle fibres (Trontelj and Stålberg, 1983), possibly due to cryptic intramuscular nerve fibre injury or an unstable muscle membrane from a hormonally mediated channelopathy. Findings in urethral sphincter EMG and maximum urethral closure pressure have been shown to correlate (Wiseman, 2002), and a pulsatile urethral pressure trace has been shown to be associated with decelerating bursts in the EMG (Sihra, 2016).

Complex repetitive discharges have also been recorded from the external anal sphincter, suggesting that there could be wider pelvic floor dysfunction in some patients (Webb, Fawcett and Neal, 1992). Complex repetitive discharges and decelerating bursts have also been recorded from the urethral sphincter of females not reporting urinary symptoms, more prevalent in the mid-luteal phase of the menstrual cycle (Ramm, 2012, Tawadros, 2015). Therefore, the EMG findings should be interpreted in the clinical context of the patient. Diagnosing Fowler’s syndrome has therapeutic implications as patients have favourable outcomes following sacral neuromodulation (De Ridder, Ost and Bruyninckx, 2007), the gold standard treatment for chronic idiopathic urinary retention in women, and botulinum toxin injected into the external urethral sphincter (Wright, 2024). Case vignette 5 illustrates a case of chronic idiopathic urinary retention and the findings from urethral sphincter EMG.

**Case vignette 5.** A young female presenting with chronic idiopathic urinary retention.

A female in her early 30 s presented with complete urinary retention after undergoing a laparoscopy for the investigation of pelvic pain. She was able to void once again after 3 days, however was experiencing urinary urge, urinary hesitancy and a sensation of incomplete bladder emptying. She was persistently retaining urine and the postvoid residual volumes were in the range of 200–300 mL and was initiated on intermittent catheterization. She did not report any significant bowel or perineal sensory complaints. MRI lumbosacral spine did not reveal any significant abnormalities. Her urodynamics study demonstrated an obstructed voiding pattern in the pressure flow study with a maximum flow rate (Qmax) of 4 ml/second (normal for this age and sex >18 ml/second) at a maximum pressure (PdetQmax) of 71 cmH20, and the post void residual volume was 200 mL. Urethral pressure profilometry revealed an elevated maximum urethral closure pressure of 101 cmH2O (expected value for this age was 60 cmH2O).

**Fig. 10:**
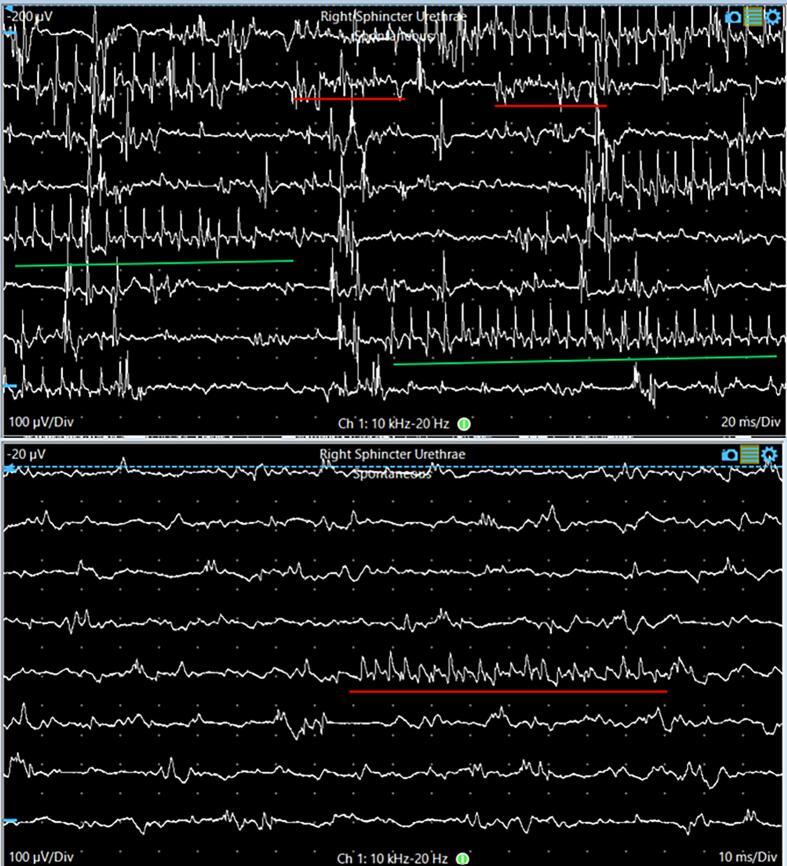
Decelerating bursts in the urethral sphincter EMG (red line) showing very small amplitude rhythmic activity, which can be appreciated mainly by the EMG sound. At times, this activity is superimposed by CRDs (green line).

She was referred for pelvic neurophysiology testing with the question whether there was any evidence for a primary disorder of urethral sphincter relaxation as seen in Fowler’s syndrome. The neurological examination was normal. Concentric needle EMG recording from the external urethral sphincter on the right side demonstrated several complex repetitive discharges and decelerating bursts (representative depiction in Fig. 10).

She was diagnosed with Fowler's syndrome and received sphincter botulinum toxin injections and became catheter free.

### 3.5 Evaluation of sexual dysfunction

Sexual function in health requires the integrity of different neural pathways and only the somatic innervation can be assessed by neurophysiology testing. Pudendal afferents carry genital sensory information to the sacral cord and play a crucial role in modulating the sexual arousal response, and abnormalities in pudendal SEPs have been demonstrated in patients with erectile dysfunction when the somatic innervation has been injured (Bemelmans, 1991, Kaiser, 2001, Tackmann et al., 1988, Tackmann et al., 1987). Abnormalities in the BCR have also been reported in men with erectile dysfunction (Bemelmans, 1991, Yang, 2000). The neurophysiology of ejaculation involves both somatic and autonomic pathways (Everaert, 2010), and anejaculation has been reported following injury to the dorsal nerve of the penis (Wieder, 2000). Neurological lesions can affect sexual functions, and BCR and pudendal SEPs allow for the interrogation of the sacral reflex arc to localise the site of lesion and to plan management of sexual dysfunction (Moon, Kang and Chun, 1993).

The severity of sexual dysfunction following cauda equina injury has been shown to correlate with neurophysiological changes in the sacral reflex arc (Podnar, Oblak and Vodušek, 2002). Erectile dysfunction in diabetic men has also been associated with abnormal findings in BCR and pudendal SEPs (Sarica, 1994, Sartucci, 1999). Additionally, pudendal SEP abnormalities have been demonstrated in men and women with multiple sclerosis (MS) reporting sexual dysfunction (Wright, et al., 2022, Yang, 2000, Yang, 2001), suggesting a lesion affecting the DCML pathway. Unilateral dorsal nerve of the penis SEP studies have been shown to identify lesions more frequently than pudendal SEP (Yang, 2000). Dynamic pudendal nerve compression from cycling can result in genital numbness and erectile dysfunction (Balasubramanian, 2020). Sexual dysfunction has been reported following myelin oligodendrocyte glycoprotein antibody-associated demyelinating disease (Hentzen, 2023, Li, 2020, Wright, et al., 2022) and with sacral Tarlov cysts (Hentzen, 2023), where abnormalities in pelvic neurophysiology tests have been reported. Small nerve fibres play an important role in sexual function, including the perception of sexual sensations, erection, and ejaculation. Assessment of these fibres may be warranted when the aetiology of sexual dysfunction is unclear (Fishel, 2000). The association between the sympathetic skin response (SSR) and sexual dysfunction remains a matter of debate (Derouet, 1995, Shahani, 1984). However, in patients with diabetes, incorporating SSR into the neurophysiological evaluation can improve the detection rate of neurological abnormalities (Bird and Hanno, 1998). Furthermore, absence of the genital SSR has been linked to sexual dysfunction in women with multiple sclerosis (Seçıl, 2007).

**Fig. 11:**
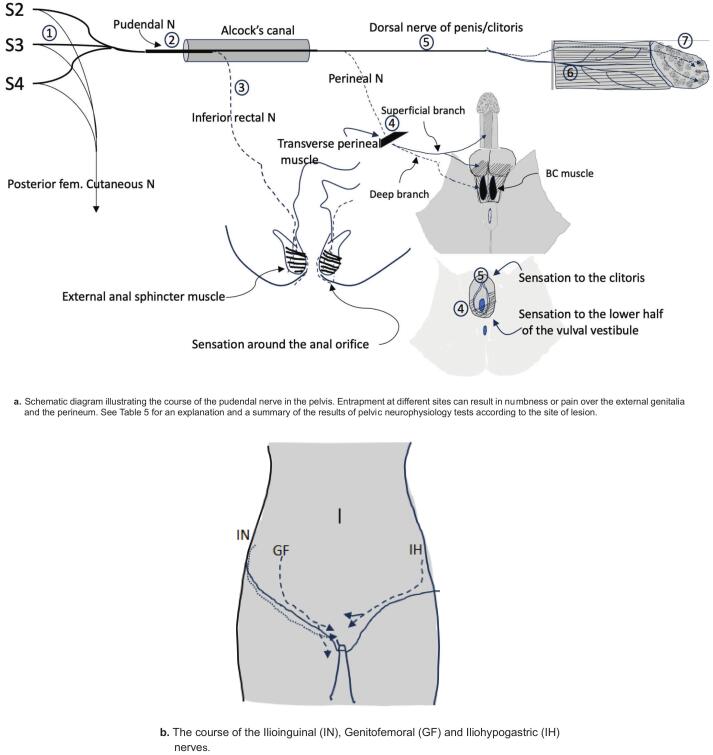
A. Schematic diagram illustrating the course of the pudendal nerve in the pelvis. Entrapment at different sites can result in numbness or pain over the external genitalia and the perineum. See Table 5 for an explanation and a summary of the results of pelvic neurophysiology tests according to the site of lesion. Fig. 11b. The course of the Ilioinguinal (IN), Genitofemoral (GF) and Iliohypogastric (IH) nerves.

Genital numbness can be reported in the context of sexual dysfunction, which could occur with central demyelinating lesions (Orasanu, 2013), spinal cord injuries (Zhang and Li, 2020), sacral root (Baker, Wilson and Wallach, 2018) or peripheral nerve lesions. Table 5 lists the different peripheral nerve lesions that can present with genital numbness. Lesions of the pudendal nerve and its branches (Stav, Dwyer and Roberts, 2009), illustrated in Fig. 11a and described in Table 5, can cause genital numbness. However ilioinguinal, iliohypogastric and genitofemoral nerves also contribute to the somatic innervation of the external genitalia and entrapment of these nerves can also present with genital numbness (illustrated in Fig. 11b and described in Table 6). Medications such as selective serotonin reuptake inhibitors (SSRIs), isotretinoin and finasteride can also be associated with genital numbness and sexual dysfunction which may persist after the medications are discontinued (Healy, 2022, Pirani, 2024). In these conditions, neurophysiology tests will be normal.

**Table 5:** Pelvic neurophysiology test findings according to the site of neurological lesion in patients presenting with pain or numbness over the external genitalia and anal areas. BC: Bulbocavernosus; BCR: Bulbocavernosus reflex; QST: Quantitative sensory testing;  − Abnormal study.

Lesion site	Site of nerve lesion	Clinical presentation	Abnormal neurophysiology tests
1	Sacral root	Genital numbness or pain, urogenital and bowel dysfunction, numbness or pain over the gluteal area and the posterior thigh. Variable weakness of lower limb muscles receiving sacral innervation. A plexus lesion can be suspected if the weakness extends to the lower limbs.	□ Tibial SEP (This can be abnormal in plexus lesion) S2 dSEP S3 dSEP S4 dSEP Pudendal SEP BCR Anal sphincter EMG
2	Pudendal nerve in the Alcock’s canal, proximal to the inferior rectal branch	Genital numbness/pain, urogenital and bowel dysfunction. No symptoms over the gluteal area or posterior thigh.	Pudendal SEP BCR Anal sphincter EMG
3	Pudendal nerve − inferior rectal branch lesion	Constipation or faecal incontinence. Pain during defecation. Numbness in the anal canal and around the external anal orifice. No symptoms over the gluteal area, posterior thigh or external genitalia.	S4 dSEP (may be abnormal) Anal sphincter EMG
4	Pudendal nerve − Perineal branch lesion	Numbness or pain over ventral penis and posterior scrotum. Numbness over the labia and vagina. Poor ejaculation and post-voiding dribbling. No symptoms over the gluteal area or posterior thigh.	Pudendal SEP- in males, dorsal stimulation will be normal and ventral stimulation abnormal; normal in females BCR BC muscle EMG
5	Pudendal nerve −lesion between the Alcock’s canal and proximal to root of the penis/ clitoris	Numbness over the penis /clitoris. Normal ejaculation or erectile dysfunction. No bowel or bladder dysfunction. No symptoms over the gluteal area or the posterior thigh.	Pudendal SEP- dorsal stimulation will be abnormal in males; abnormal in females BCR QST
6	Pudendal nerve- dorsal penile branch lesion	Unilateral numbness or pain over the shaft of the penis. No bowel or bladder dysfunction. No symptoms over the gluteal area or posterior thigh.	Dorsal nerve of penis conduction study
7	Small fibre neuropathy affecting the external genitalia	Numbness or pain over the tip of the penis/clitoris. Sexual dysfunction, but no ejaculation issue.	QST

**Table 6:** The distribution of genital numbness or pelvic pain following injury to peripheral nerves innervating the external genitalia.

Nerve	Branch	Pubis	Clitoris	Labia majora	Labia minora	Vagina	Perineum /anal	Scrotum	Root penis	Shaft	Glans penis
Iliohypogastric nerve	Lateral cutaneous (iliac) branch	+									
Ilioinguinal nerve	Anterior Labial/scrotal	+	+	Anterior 1/3rd				Anterior 1/3rd	+		
Genitofemoral nerve	Genital branch	+		Anterior				Anterior			
Pudendal nerve	Inferior rectal						Anal canal & anal orifice				
	Superficial perineal			Posterior 1/5th	+	Lower 1/5th		Posterior			
	Dep perineal						External urethral sphincter/ urethra afferents				
	Dorsal nerve of penis/clitoris									+	+
Posterior femoral cutaneous nerve	Perineal branch(Jiamjunyasiri et al., 2023)		+	+			Lateral part	Anterior			

Case vignette 6 illustrates a case of sexual dysfunction in a male patient.

**Case vignette 6.** Case of sexual dysfunction in a male

A male in his 40 s presented with 3 years of progressive erectile dysfunction and genital numbness which started at the root of the penis and extended to the shaft and anterior scrotum. Sensations were normal over the glans. Other sexual domains were unaffected, and he did not report bladder or bowel symptoms. MRI lumbosacral spine showed only minor degenerative changes in the spine without any evidence for conus or cauda equina compression.

**Fig. 12:**
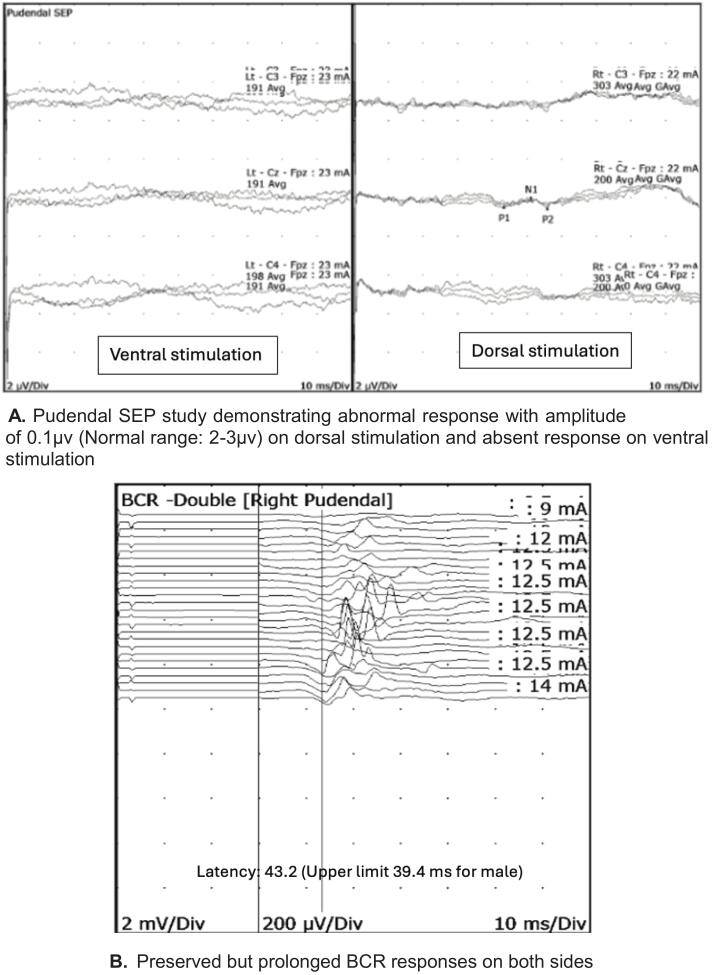
A. Pudendal SEP study demonstrating abnormal response with amplitude of 0.1µv (Normal range: 2-3µv) on dorsal stimulation and absent response on ventral stimulation Fig. 12B. Preserved but prolonged BCR responses on both sides.

He was referred for pelvic neurophysiology testing with the question whether there was a lesion affecting the pudendal nerve or sacral roots. The neurological examination demonstrated a reduction in sensation to pinprick over the penis and anterior scrotum, and to light touch assessed using von Frey monofilament hairs on the ventral surface of the penis. The rest of the examination was unremarkable. Neurophysiology tests were abnormal (Table 7) and findings in the pudendal SEP and BCR tests are illustrated in Fig. 12A and 12B, respectively. These findings confirmed a partial pudendal nerve lesion affecting the sensory fibres. MRI neurography study of the pelvis was normal and did not demonstrate entrapment of the pudendal nerve.

**Table 7:** Summary of pelvic neurophysiology findings in case vignette 6.

Test	Right	Left
Tibial	Normal	Normal
S2 dSEP	Normal	Normal
S3 dSEP	Normal	Normal
S4 dSEP	Normal	Normal
Pudendal	Prolonged latency (dorsal) Absent (ventral)	
BCR	Abnormal	Abnormal
Dorsal nerve of penis sensory study	Normal	
Anal sphincter EMG	Normal	Normal

Case vignette 7 illustrates a case of genital numbness in a female patient.

**Case vignette 7.** Case of genital numbness in a female.

A female in her mid-50s presented with genital numbness of 8 years duration. Symptoms began acutely following prolonged sitting on a static bicycle in the gym and the numbness was noticeable over the left side of the vulva involving the clitoris and labia. Numbness would worsen when sitting on hard seats, however at no point did she ever experience pain in the perineum. Sensations elsewhere were normal. MRI lumbosacral spine showed a central disc protrusion at L5/S1 level with a caudal migration compressing the left S2 root.

**Fig. 13:**
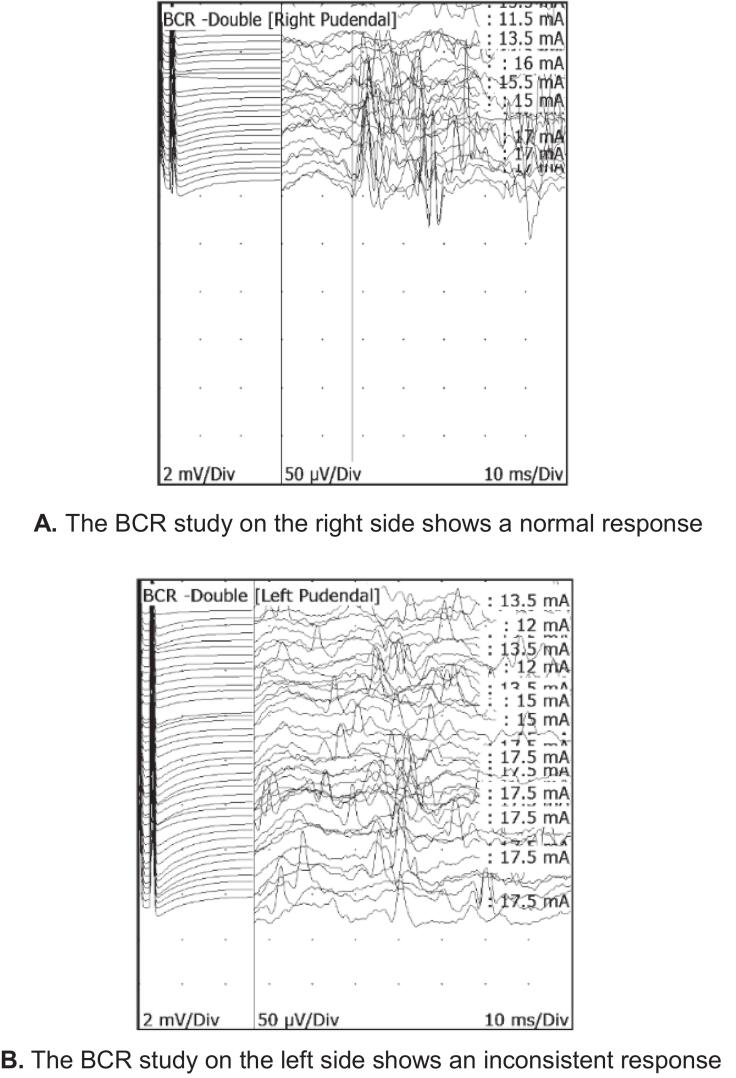
A. The BCR study on the right side shows a normal response Fig. 13B. The BCR study on the left side shows an inconsistent response.

An S2 root lesion could not explain her symptoms, and she was therefore referred for pelvic neurophysiology testing with the question whether there was any evidence for pudendal nerve injury or an S4 root injury. The neurological examination demonstrated a reduction in sensation to pinprick to 20% over the clitoris and the left labia minora. Light touch assessed using von Frey monofilament hairs was normal. The rest of the examination was unremarkable. The tests were abnormal (Table 8) and findings in the BCR test are illustrated in Fig. 13A and 13B.

**Table 8:** Summary of pelvic neurophysiology findings in case vignette 7.

Test	Right	Left
Tibial SEP	Normal	Normal
S2 dSEP	Normal	Normal
S3 dSEP	Normal	Normal
S4 dSEP	Normal	Absent
Pudendal SEP	Normal	Absent
BCR	Normal	Inconsistent
Anal sphincter EMG	Normal	Normal

These findings confirmed a partial injury of the left side pudendal nerve, with likely localisation distal to the inferior rectal branch. MRI neurography study of the pelvis was normal and did not demonstrate entrapment of the pudendal nerve.

### 3.6 Evaluation of chronic pelvic pain

**Fig. 14:**
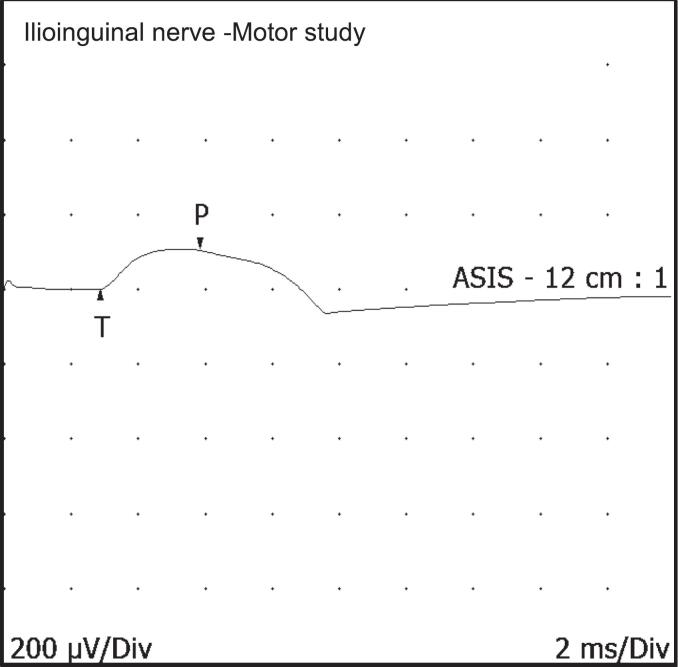
Ilioinguinal nerve motor conduction study (Latency,2.9 ms; amplitude,0.1 mV)**.** The reference electrode is placed 2 cm proximal to the pubic symphysis, and the active electrode is placed 2 cm laterally on the ipsilateral side (Cho et al., 2017). The stimulation site is at the inguinal ligament medial to the anterior superior iliac spine. Prolonged latency with reduced amplitude or absent response is considered abnormal.

The term pudendal neuralgia is often used synonymously with chronic pelvic pain. However, chronic pelvic pain can result from involvement of a number of different nerves such as the ilioinguinal (Wong and Ng, 2020), genitofemoral (Cesmebasi, 2015), posterior femoral cutaneous (Williams, 2020) or iliohypogastric (Kohan, McKenna and Irwin, 2020) nerves. Pain can also originate from the pelvic viscera when stretched or overdistended from signalling through the visceral afferent pathways (Lamvu and Steege, 2006). In addition, visceral or dermatomal afferent signals can influence somatic efferent pathways at the spinal cord through cross-organ sensitisation (Ustinova, Fraser and Pezzone, 2006). The ilioinguinal nerve exits the inguinal canal and injury can result in pain over the anterior labia /scrotum. The perineal branch of the pudendal nerve is responsible for sensations over the medial labia, whereas the dorsal clitoris/ penile nerve is responsible for sensations over the clitoris /penis, and the perineal branch of the posterior cutaneous nerve is responsible for sensations over the one fifth of the labia majora (Weber, Vilensky and Carmichael, 2018). Injuries to these nerves may present as chronic pelvic pain in the respective nerve territories. Table 5 lists the different peripheral nerve lesions that can present with genital pain. Peripheral nerve entrapment can present with genital pain (illustrated in Fig. 11a and 11b, and described in Table 6). Injury to the inferior rectal nerve can present with perianal pain (Table 5, Table 6). Ilioinguinal nerve damage can also present with genital pain, mainly at the root of the penis or clitoris, scrotum /labia and upper medial thigh. The nerve mainly originates from the spinal nerve L1 and emerges at the Anterior Superior Iliac Spine (ASIS). It pierces the transversus abdominis muscle and travels through the inguinal canal, and gives off its terminal sensory branches to the genital region. Normal motor conduction is demonstrated in Fig. 14. The nerve can be damaged following surgical incisions or suture entrapment, resulting in pain in the genital region which can be mistaken as clitoral pain.

Dysfunction of any of these nerves can result in non-specific debilitating pain that can often be mistaken as pudendal neuralgia and testing of the pudendal nerve alone may miss the nerve injury. A thorough clinical examination is essential to map out the distribution of pain in the distribution of a dermatome or nerve territory. Case vignette 8 illustrates a case of pudendal neuralgia.

**Case vignette 8.** Case of pudendal neuralgia.

A female in her 70 s presented with pain in the perineum that started around 4 years prior. She described the pain as having a “twisting” quality, similar to a spasm, occurring in relation to the opening of the vagina and back passage which extended into the vagina and rectum. The pain would become worse within minutes of sitting down, though she did not experience pain when seated on the toilet. She was being treated for atrophic vulvovaginitis. MRI of the thoracolumbar spine revealed a mid-thoracic cord syrinx and no enhancement was noted with gadolinium administration.

**Fig. 15:**
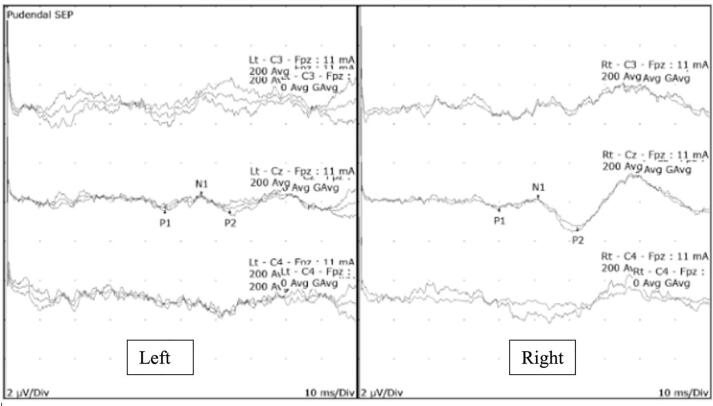
Pudendal SEP study demonstrating a prolonged latency more than 6 ms on the left compared to the right.

She was referred for pelvic neurophysiology testing with the question whether the syrinx was compressing the sacral fibres within the dorsal column. Her symptoms were typical for pudendal neuralgia, and a second question was whether there was any evidence for pudendal nerve injury. The pelvic neurological examination demonstrated patchy, inconsistent sensory impairment over the S2, S3 and S4 dermatomes bilaterally. The neurophysiology tests were abnormal (Table 9) and findings of the left pudendal SEP test are illustrated in Fig. 15. These findings confirmed an injury of the left side pudendal nerve affecting the sensory and motor fibres. MRI neurography study of the pelvis was normal and did not demonstrate entrapment of the pudendal nerve. There was no neurophysiological evidence of compression of the dorsal column by the syrinx.

**Table 9:** Summary of pelvic neurophysiology findings in case vignette 8.

Test	Right	Left
Tibial SEP	Normal	Normal
S2 dSEP	Normal	Normal
S3 dSEP	Normal	Normal
S4 dSEP	Normal	Normal
Pudendal SEP	Normal	Abnormal
Anal sphincter EMG	Normal	Mildly abnormal

### 3.7 Evaluation of voiding dysfunction

Urodynamics testing is designed to investigate lower urinary tract symptoms to evaluate how well the bladder and urethral sphincter function during bladder filling and emptying. Kinesiologic EMG recording performed concomitantly with urodynamic testing has been used to assess sphincter activity, particularly to recognise detrusor-sphincter dyssynergia (DSD). This is a characteristic abnormality seen in patients reporting lower urinary tract dysfunction following SCI where there is concomitant contraction of the detrusor and external urethral sphincter during attempted voiding. Detrusor-sphincter dyssynergia is an important cause of voiding dysfunction and urinary retention (Panicker, Fowler and Kessler, 2015) and is important to recognise, being a risk factor for developing renal impairment (Ginsberg, 2021).

In health, EMG activity in the external urethral sphincter ceases during voiding, whereas in patients with DSD, EMG activity persists or increases due to increased recruitment of motor units during attempted voiding (Furrer, Kessler and Panicker, 2024). Early studies introduced external urethral sphincter concentric needle recording into urodynamics practice and defined DSD as increased EMG activity during a detrusor contraction (Blaivas and Fisher, 1981). Inappropriate contraction or failure of relaxation of either the internal (smooth muscle) and/or external (striated muscle) urethral sphincter concomitant with detrusor contraction was demonstrated with simultaneous recording of external urethral sphincter activity by EMG (Yalla, 1977). EMG activity was also recorded from the external anal sphincter using monopolar needles, and DSD was defined as increased anal sphincter activity when instructed to void or perform a Valsalva manoeuvre (Mayo, 1979). However a similar pattern of sphincter activity mimicking DSD can be recorded with voluntary contraction of the external sphincter by uncooperative patients, or in case of learned abnormal behaviours in patients presenting with dysfunctional voiding such as in Hinman’s syndrome with a non-neurogenic neurogenic bladder (Groutz, 2001). Using a concentric needle can be uncomfortable for patients, and therefore surface electrodes are generally preferred. However, the amplitude of EMG activity recorded from surface electrodes is smaller because of interposed adipose tissue, and the signal quality can be contaminated by artifacts from 50 Hz electromagnetic interference and electrode displacement from insufficient skin adhesion due to moisture or, in females, the urinary stream tracking down the perineum (Spettel, Kalorin and De, 2011). The International Continence Society (ICS) guidelines on urodynamic equipment standardisation recommend that EMG recordings during urodynamics should adhere to specific criteria, including a high input impedance exceeding 100 megaohms, a common-mode rejection ratio (CMRR) greater than 80 and the incorporation of a filtering program. These specifications aim to optimise the consistency of EMG recordings during the testing (Gammie, 2014). However despite this, published statements regarding EMG technique and reporting vary considerably. This is partly due to the evolving definition of DSD, and because the established neurophysiology techniques which are nowadays used for diagnosing DSD have not been adapted to modern-day consensus definitions. Nowadays, the diagnosis of DSD is increasingly being established by fluoroscopy performed during urodynamic testing (videourodynamics testing), however kinesiologic EMG recording still has a role in centres where facilities for performing such urodynamics studies are lacking.

## 4 Conclusion

Pelvic neurophysiology testing offers a unique means of assessing the segmental somatic innervation of the sacral spinal cord (S2–S4), and uncover patterns of nerve injury not apparent through imaging, laboratory studies and other functional tests alone. Given the complexity and invasive nature of some of the tests, they should be performed with a clear clinical question that guides test selection and interpretation. These tests provide objective evidence of nerve dysfunction and are particularly valuable in specific scenarios, such as the evaluation of suspected cauda equina syndrome, MSA, chronic urinary retention, pelvic pain, sexual dysfunction and situations where MRI findings are inconclusive. When used as an adjunct to clinical examination, neurophysiological testing enhances diagnostic accuracy in the assessment of patients with neuro-urological disorders.

## CRediT authorship contribution statement

**Jalesh N. Panicker:** Conceptualization, Methodology, Project administration, Formal analysis, Writing – original draft, Writing – review & editing. **Sara Simeoni:** Conceptualization, Methodology, Formal analysis, Writing – original draft, Writing – review & editing. **Sarah Wright:** Writing – original draft, Writing – review & editing. **Claire Hentzen:** Writing – original draft, Writing – review & editing. **Prasad Malladi:** Conceptualization, Methodology, Formal analysis, Writing – original draft, Writing – review & editing.

## Declaration of competing interest

The authors declare that they have no known competing financial interests or personal relationships that could have appeared to influence the work reported in this paper.
